# Antioxidant Bioactivity of Samsum Ant (*Pachycondyla sennaarensis*) Venom Protects against CCL_4_-Induced Nephrotoxicity in Mice

**DOI:** 10.1155/2014/763061

**Published:** 2014-04-03

**Authors:** Hossam Ebaid, Jameel Al-Tamimi, Iftekhar Hassan, Ibrahim Alhazza, Mohamed Al-Khalifa

**Affiliations:** ^1^Department of Zoology, College of Science, King Saud University, P.O. Box 2455, Riyadh 11451, Saudi Arabia; ^2^Department of Zoology, Faculty of Science, El-Minia University, El-Minia 61591, Egypt

## Abstract

To assess whether SAV could influence the effects of carbon tetrachloride (CCL_4_) exposure, mice were treated with SAV in doses of 100, 200, 300 and 400 **μ**g/kg body weight and the effects on oxidative status and kidney function were studied. Serum levels of creatinine, malondialdehyde (MDA), and blood urea, together with renal and hepatic levels of MDA, glutathione (GSH), superoxide dismutase (SOD), and catalase (CAT) were quantified in order to evaluate antioxidant activity. Results showed that the group injected with CCL_4_ exhibited significantly higher levels of oxidative stress markers, MDA, and significantly lower concentrations of GSH, SOD and catalase. SAV was found to significantly improve these oxidative markers, occasionally, in a dose-dependent manner. Furthermore, treatment with SAV was associated with the same behaviour in respect to kidney functions which had previously been impaired by CCL_4_. Histopathological examination demonstrated that SAV, in different groups, improved the renal tissue damage induced by CCL_4_ and histological scores confirmed that significant improvements were obtained after treatment with SAV, particularly with the lowest dose (100 **μ**g/kg body weight). In conclusion, SAV has the potential capability to restore oxidative stability and to improve kidney functions after CCL_4_ acute injury.

## 1. Introduction

Ants have been called “chemical factories” [1–3] since the venom of some ants may contain as many as 75 different components [4]. The genus* Pachycondyla* is a large group of ants mostly found in tropical and subtropical regions, and includes the Samsum ant (*P. sennaarensis*). Samsum ant is responsible for cases of anaphylaxis across the globe [5]. Human envenomation caused by these social insects generally results in pain, local inflammation, itching, and irritation, but sometimes leads to serious allergic reactions, which are trigged by a structurally diverse mix of compounds [6, 7].

Animal venoms are complex mixtures containing a range of bioactive elements, some of which have potential therapeutic uses [8] due to their high specificity and potency to act on molecular targets [9]. The aqueous solutions of proteinaceous venoms produced by ants contain enzymatic and nonenzymatic proteins, free amino-acids, and small biologically active compounds like histamine, 5-hydroxytryptamine, acetylcholine, norepinephrine, and dopamine [10]. It has been reported that the venom from* P. Sennaarensis* has a significant antitumour effect on breast cancer cells in a dose and time dependent manner without affecting the viability of nontumour cells [11, 12]. Furthermore, several studies of ant venoms have confirmed the intrinsic beneficial properties such as reduction of inflammation, pain relief, and improved function of the immune system and liver [13, 14].

## 2. Materials and Methods

### 2.1. Chemicals

Succinicacid, potassium dihydrogen and monohydrogen phosphate, glycine, pyrogallol, hydrogen peroxide, trichloroacetic acid (TCA) and ethylenediaminetetra-acetic acid (EDTA), sulphosalicylic acid, and thiobarbituric acid (TBA) were purchased from BDH, England. 5,5-dithiobis-2-nitrobenzoic acid (DTNB), dihydrogen phosphate, trichloroacetic acid, carbon tetrachloride, and thiobarbituric acid were purchased from Merck Company, Darmstadt. Melatonin was obtained from MP Biomedicals, LLC., France. Folic acid was purchased from Sigma Chemical Company, USA. The rest of all other chemicals used were of analytical grade.

### 2.2. Collection of the Samsum Ant Dissection of the Venom Gland

Colonies of* P. Sennaarensis* (containing 2000–2500 workers, with brood of all stages and multiple queens (3–8)) were collected from Al Ehsaa Governorate, East Riyadh, and the Kingdom of Saudi Arabia. Collected nests were moved to the ant insectary in the Zoology Department, College of Sciences, King Saud University. The ants were housed in plastic nest-bottles within a large plastic box (45 × 30 × 18 cm) until venom extraction. The sting apparatus was removed by grabbing the last segment of the abdomen and detaching it with the sting apparatus. The venom gland was pinched out and placed in a small tube [15]. Glands were homogenized, and then centrifuged at 1000 rpm for 2 min. and the supernatant was collected, lyophilized, and kept under −20°C.

### 2.3. Ethical Approval

This study did not involve endangered or protected species. Regarding experimental animals, all procedures were conducted in accordance with the standards set forth in the guidelines for the care and use of experimental animals by the Committee for the Purpose of Control and Supervision of Experiments on Animals and the National Institutes of Health. The study protocol (care and handling of experimental animals) was approved by the Animal Ethics Committee of the Zoology Department in the College of Science at King Saud University.

### 2.4. Experimental Design

Fourty-eight male mice were divided into six groups, eight mice each. The first was the untreated negative control group. Group 2 received a single dose of 1 mL/kg CCl_4_ in liquid paraffin (1 : 1 volume) through an intraperitoneal (IP) injection [16]. Groups 3–6 were pretreated with a dose of 100, 200, 300, and 400 *μ*g/kg SAV, respectively, three times with two days intervals via IP route. These four groups were then injected with a single dose of 1 mL/kg CCl_4_ in liquid paraffin (1 : 1 volume) through an IP injection for challenge.

### 2.5. Estimation of Creatinine and Urea as Kidney Function Markers

The level of creatinine and urea was estimated in the serum by the commercially available diagnostic kits (Quimica Clinica Aplicada S.A., Spain).

### 2.6. Assay of Antioxidant Enzymes (SOD and CAT)

The activity of different antioxidant enzymes was assayed with standard protocols. Cu Zn superoxide dismutase (CuZnSOD) was assayed by autoxidation of pyrogallol [17] while that of catalase (CAT) was done by decomposition of hydrogen peroxide [18].

### 2.7. Estimation of GSH Level

The level of reduced glutathione (GSH) was estimated by method of Jollow et al. [19].

### 2.8. Estimation of MDA Level

The extent of lipid peroxidation was estimated by the method of Buege and Aust [20] involving the measurement of total malondialdehyde (MDA).

### 2.9. Histological Sections

Kidney parts were collected from the sacrificed control and different treated mice groups. Tissues were fixed in Bouin's fixative, processed into paraffin, and 4 micrometer thick sections were prepared. Sections were stained with Haematoxylin and Eosin (H&E) for general histological architecture. In each group, many sections from different mice were investigated and the clear and common changes were photographed. Histopathological changes were scored according to Dommels et al. [21]. A rating score between (−) (no change) and (+++) was assigned for each investigated section. Sections from at least five mice were carefully investigated.

### 2.10. Statistical Analysis

All the data have been expressed as in mean ± standard error of mean (SEM) for 5-6 different preparations in duplicate. Their statistical significance was evaluated by one-way ANOVA and Tukey's post hoc analysis by “GraphPad Prism 5.” The probability of occurrence was selected at *P* ≤ 0.05. The treatment and the experiments were repeated twice to check reproducibility of the results.

## 3. Results

### 3.1. Effect of Samsum Ant Venom on Kidney Function Markers

**Figure 1:**
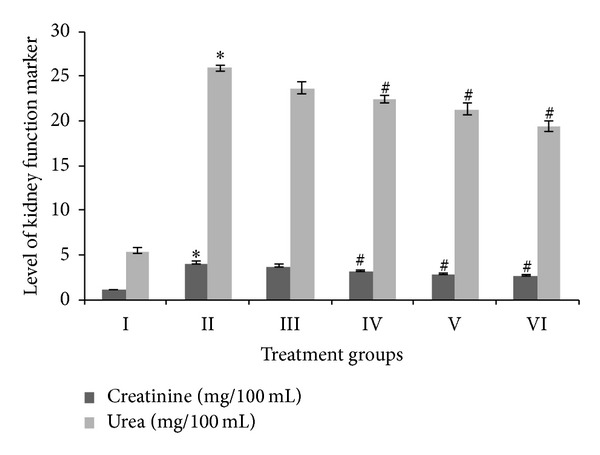
Levels of urea and creatinine as kidney function markers, expressed in milligrams per 100 milliliters of the serum samples. ∗ indicates significantly different from control. # indicates significantly different from group II.

In the present study, levels of creatinine and urea in serum were chosen as kidney function markers. The CCl_4_ treated group II showed creatinine levels increasing by 271% as compared to the control. Meanwhile, all the SAV treated groups showed dose dependent decreases in the level of creatinine compared to group II, with groups III, IV, V, and VI exhibiting a decrease in creatinine of 7.6%, 20.3%, 28.67%, and 32%, respectively, compared to group II (Figure 1). Group II also displayed a staggering increase in the level of urea, that is, by 376.97%, compared to the control. Groups III, IV, V, and VI, meanwhile, showed decreases in urea level by 8.65%, 13.32%, 17.83%, and 25.32% with respect to group II.

### 3.2. Effect of Samsum Ant Venom on Renal Structure

**Figure 2:**
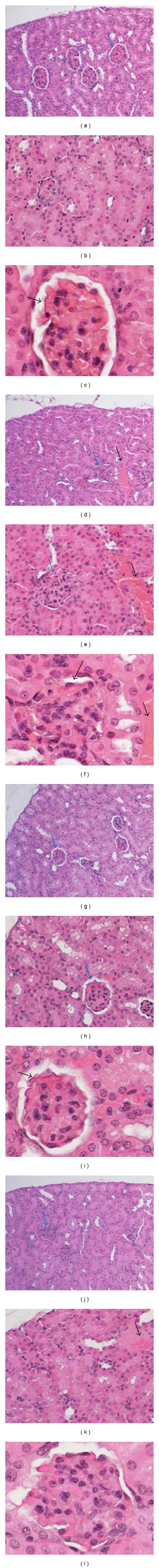
Representative renal tissue stained with H&E. The control renal tissue shows the normal architecture of the kidney cortex showing the glomeruli (blue arrow) ((a); ×200), tubules ((b); ×400), and the normal urinary space ((c); ×1000). The histopathological changes in the CCL_4_ injected mice showing oedematous glomeruli (blue arrows), a haemorrhage (thick black arrow) ((d); ×200, (e); ×400), and a narrow urinary space (thin black arrow) with a further haemorrhage (thick black arrow) ((f); ×1000). Changes in the renal tissues in the SAV treated mice (100 *μ*g/kg) after CCL_4_ injection show more normal glomeruli (blue arrows) ((g); ×200, (h); ×400) and urinary space (thin black arrow) similar to that of the control mice ((i); ×1000). Changes in the renal tissues in the SAV treated mice (300 *μ*g/kg) after CCL_4_ injection show narrow urinary spaces in glomeruli (blue arrows) ((j); ×200), haemorrhage in a dilated blood vessel ((k); ×400), and glomerulus ((l); ×1000).

Normal renal tissues of mice are presented in Figure 2. Histopathological examination of the renal tissues of the CCL_4_ treated mice, however, showed that some lumens were hyaline with intensive haemorrhaging in the blood vessels, which appeared dilated. Histological examination of renal sections from the same group also showed narrow urinary spaces which may be due to oedema. In addition, some blood vessels were detected inside the glomerulus with obviously disturbed cells in the collecting duct wall. On the other hand, an overall improvement in the renal architecture was observed after treatment with SAV with many tissue damage markers being recovered. Nonetheless, after SAV treatment, some glomeruli still appeared shrunken and there remained some detectable haemorrhage (Figure 2).

Histological scores confirmed that an improvement took place after treatment with SAV, in particular at the lower dose of 100 *μ*g/kg body weight. Although the SAV treated mice appeared structurally improved at the higher doses (200–400 *μ*g/kg body weight), some histopathological signs were obviously detected (Table 1), including the presence of inflammatory cells, haemorrhaging, and eosinophilic hyaline casts, especially in the CCL_4_ group treated with a dose of 400 *μ*g/kg body weight.

### 3.3. Effect of Samsum Ant Venom on Antioxidant Status

**Figure 3:**
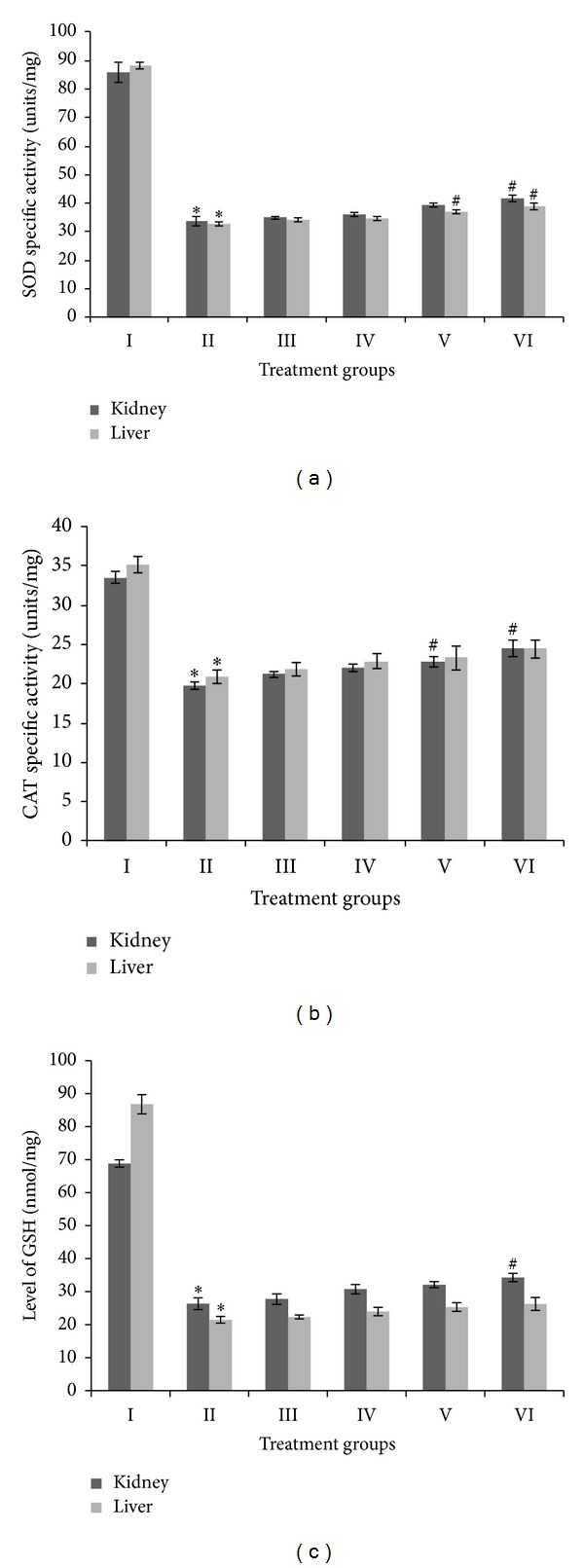
Level of specific activity of superoxide dismutase (SOD) in kidney and liver samples, expressed in enzyme units per milligram of protein (a), level of specific activity of catalase (CAT), expressed in enzyme units per milligram of protein (b), and level of reduced glutathione (GSH), expressed in enzyme nanomoles per milligram of protein (c). ∗ indicates significantly different from control. # indicates significantly different from group II.

In the present study, superoxide dismutase (SOD), catalase (CAT), and reduced glutathione (GSH) were taken as major parameters to assess the antioxidant status in the major organs of the kidney and liver of the treated animals. Group II showed a striking decline in the specific activity of SOD, by 60.8% and 63.15% in kidney and liver samples, as compared to their controls. Groups III, IV, V, and VI, however, showed increases in SOD activity, compared to group II, of 4.02%, 7.17%, 16.75%, and 24.13%, respectively, in the case of the kidney samples, and by 4.85%, 6.35%, 13.48%, and 19.13% in the case of the liver samples. These results indicate that the extent of improvement in SOD activity due to SAV is obviously marked in the kidney (Figure 3(a)).

Group II demonstrated a decrease in CAT specific activity by 40.88% in the kidney and 40.43% in the liver, compared to the respective controls. In the SAV and CCL_4_ treated groups, groups III, IV, V, and VI showed improvement in CAT activity by 7%, 11.55%, 15.23%, and 23.96% in kidney samples and by 4.39%, 9.32%, 11.46%, and 17.1% in the liver samples, respectively, compared to group II (Figure 3(b)).

The kidney samples of group II showed a decrease in GSH level by 61.72% compared to the control while groups III, IV, V, and VI displayed improvements in this level by 5.24%, 16.77%, 21.93%, and 30.09%, respectively, compared to group II. Group II liver samples, meanwhile, demonstrated a decrease in the GSH level by 75.27% compared to the control, while groups III, IV, V, and VI showed increases by 3.96%, 11.7%, 18.08%, and 22.41%, respectively (Figure 3(c)).

### 3.4. Effect on MDA Level

**Figure 4:**
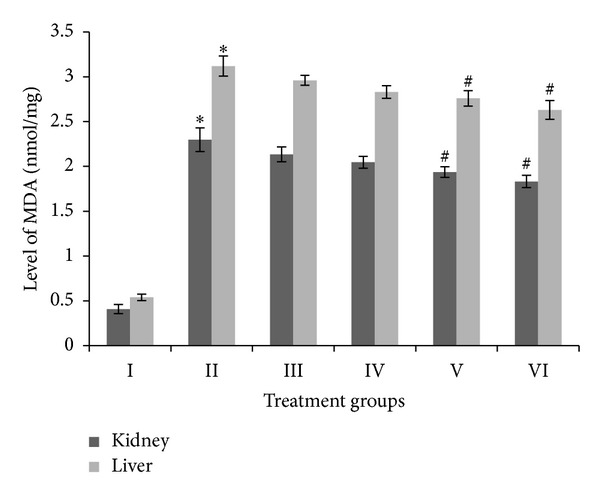
Level of malondialdehyde (MDA), expressed in enzyme nanomoles per milligram of kidney and liver samples. ∗ indicates significantly different from control. # indicates significantly different from group II.

MDA is the first stable product of lipid peroxidation and is a good parameter to assess oxidative stress in treated animals. In the present study, it was estimated in liver and kidney samples. Group II showed a very sharp increase in the level of MDA, that is, of 461.85% in kidney and 477.78% in liver samples, compared to their respective controls. Groups III, IV, V, and VI showed moderate decrease of 7.14%, 10.96%, 15.75%, and 20.28%, respectively, in kidney samples, and of 5.12%, 9.23%, 11.54%, and 16.02% in liver samples (Figure 4).

## 4. Discussion

Many animal products have been shown to have potential therapeutic effects [8, 22, 23] due to their high specificity and potency in acting on molecular targets [9, 10]. The ant venom has a significant antitumour effect [11, 12] and provides pain relief, a reduction in inflammation, and improved function of the immune system and liver [13, 14]. Here, we evaluated the efficacy of SAV, a potent antioxidant and free-radical scavenger, on CCL_4_ induced renal damage and oxidative stress in experimentally intoxicated mice. Our results indicate that SAV exerts a protective effect against CCL_4_ nephrotoxicity, when applied in a range of doses. A higher dose of SAV, however, may be able to optimize the antioxidant system in mice but is not effective enough to show structural improvement. Lower doses of SAV, meanwhile, appeared to be more effective and it is also possible that the antioxidant optimization required to remove structural aberrations in the tissues is achieved at these lower doses.

SAV was found to be effective in replenishing the activity of vital antioxidant enzymes and proteins like SOD, CAT, and reduced glutathione in CCl_4_ pretreated mice, although the effect was significant only at higher doses (400 *μ*g/kg). In addition, while key parameters of kidney function markers (urea and creatinine) were found to be elevated in CCl_4_ pretreated mice, the levels of these markers tended towards normal values with increasing dose of SAV.

A relationship between nephrotoxicity and oxidative stress has been demonstrated in many experimental models [24] and the results in our study in relation to lipid peroxidation demonstrated the same pattern as the organ function markers.

Previously, [25] it has been found that SAV has immunity and antioxidant boosting properties and the results of this study confirm this earlier work by demonstrating that CCl_4_ induced oxidative stress in mice can be countered by treatment with SAV in appropriate doses. The findings from the present investigation are also in accordance with previous reports that show that oxidative stress plays an active role in the pathogenesis of the majority of toxicants, pollutant, and drugs, both in vitro and in vivo [26–28].

The kidney is a common target for toxicity since its large share of blood flow gives it the capacity to extract and concentrate toxic substances [28]. The glomerular hypertrophy in CCL_4_ injected mice may be due to the proliferation of mesangial cells, which secrete more matrixes. Furthermore, the blood capillaries appeared engorged with red blood cells and the urinary space was completely obliterated as previously found with indomethacin injection [29] and with piroxicam injection [30]. The tubular lesions observed in the present study were accompanied by an invasion of inflammatory cells into the intertubular tissues in an attempt to minimize the injury. Some of these external stressors apparently caused the tubular lesions. In the present study, the different segments of the loop of Henle were less affected by the CCL_4_ dose which may suggest that its main targets are the convoluted and collecting tubules. Jaekson and Lawrence [31] found that treatment with either indomethacin or phenylbutazone caused papillary necrosis, tubular degradation, and inflammatory cellular infiltration, and these results agree with our own.

On the other hand, statistical analysis of the histological scores confirmed a significant improvement in the renal tissue of CCL_4_ mice after treatment with SAV. Ebaid et al. [25] have demonstrated that SAV, at the dose of 100 *μ*g/kg body weight, maintained and restored anti-inflammatory and hypolipidemic bioactivity in mice following the disruption of these parameters by LPS injection. This improvement by SAV was mediated by the upregulation of AKT1 and the induction of oxidative stability. Furthermore, they found that SAV has the capability to inhibit the gene expression of TNF-*α*, which, together with its Fas cell death-receptors, is one of the inflammatory cytokines that are central to cell destruction. SAV has also been found to have a significant antitumor effect [11, 12]. In addition, Kou et al. [13] found analgesic and anti-inflammatory activities both in the total extracted venom and in individual fractions of the venom of Chinese medicinal ants. These anti-inflammatory activities of SAV have been confirmed [32].

Taken together, the data from this work confirms the role of SAV in an overall improvement of the histological structure of renal tissues and in the reestablishment of balance following treatment with CCL_4_. In general, the differences between the SAV-treated groups were not significant and could only really be demonstrated through structural examination of the renal tissue, which showed that a higher dose of SAV caused tissue damage with diarrhoea (data not shown). Overall, the application of a low dose on three occasions with two day intervals resulted in the best outcomes from the point of view of the structure of the renal tissue, with a partial nut not complete recovery. Thus, the low dose may be applied for a longer time in order to achieve a complete structural and functional recovery. It is important to report that low doses of SAV might help the rodents to adapt and optimize their physiological systems, as is evident from the histopathological evaluation in the present study. However, although SAV shows a dose dependent replenishment in the oxidative stress parameters, this might only be true for a short duration and might be reversed if a high dose was given for a longer time. Further investigation is needed, therefore, to find out the exact reason behind these contradictory effects of SAV in relation to oxidative stress markers and histopathological results. Work dealing with the effects of different SAV doses on the intestine has also been initiated in our lab.

## Figures and Tables

**Table 1:** Histological score of the pathological changes in the renal tissues in mice treated just with CCL_4_ and those treated with CCL_4_ and different doses of SAV.

Histopathological lesions	Oedematous glomeruli	Haemorrhage	Infiltration of inflammatory cells	Hyaline casts	Disintegrated nucleus
Control	−	−	−	−	−
CCl_4_-mice	+++	+++	+++	+++	+++
100 µg/kg SAV + CCl_4_	−	−	+	−	−
200 µg/kg SAV + CCl_4_	−	−	+	−	−
300 µg/kg SAV + CCl_4_	−	+	+	−	+
400 µg/kg SAV + CCl_4_	+	+	+	−	+

−: a lack of structural changes; +: slight structural changes; ++: moderate structural changes; +++: severe structural changes.

## Abbreviations

(DTNB): 5,5-Dithiobis-2-nitrobenzoic acid
(CAT): Catalase
(EDTA): Ethylenediaminetetra-acetic acid
(GSH): Glutathione
(MDA): Malondialdehyde
(SAV): Samsum ant venom
(SOD): Superoxide dismutase
(TBA): Thiobarbituric acid
(TCA): Trichloroacetic acid.
